# Profile and Predictors of Maternal Quality of Life During Physiological Pregnancy: A Cross-Sectional Analysis

**DOI:** 10.3389/fpubh.2021.801035

**Published:** 2022-01-17

**Authors:** Rabia Ishaq, Maryam Shoaib, Nosheen Sikander Baloch, Abdul Sadiq, Abdul Raziq, Zil e Huma, Shanaz Raza, Fakhra Batool, Sajjad Haider, Fahad Saleem, Nafees Ahmad, Qaiser Iqbal, Amer Hayat Khan

**Affiliations:** ^1^Faculty of Pharmacy & Health Sciences, University of Baluchistan, Quetta, Pakistan; ^2^Department of Gynecology and Obstetrics, Sandeman Provincial Hospital, Quetta, Pakistan; ^3^Department of Gynecology and Obstetrics, Bolan Medical College, Quetta, Pakistan; ^4^Department of Biochemistry, Jhalawan Medical College Khuzdar, Khuzdar, Pakistan; ^5^Department of Statistics, University of Baluchistan, Quetta, Pakistan; ^6^Department of Zoology, Sardar Bahadur Khan Women's University, Quetta, Pakistan; ^7^Department of Pharmacy, Sardar Bahadur Khan Women's University, Quetta, Pakistan; ^8^School of Pharmaceutical Sciences, Universiti Sains Malaysia, Penang, Malaysia

**Keywords:** profile, predictors, maternal Quality of Life, physiological pregnancy, cross-sectional analysis

## Abstract

**Background:**

Quality of Life (QoL) and its determinants are significant in all stages of life, including pregnancy. The physical and emotional changes during pregnancy affect the QoL of pregnant women, affecting both maternal and infant health. Hence, assessing the QoL of pregnant women is gaining interest in literature. We, therefore, aimed to describe the QoL of pregnant women during physiological pregnancy and to identify its associated predictors in women attending a public healthcare institute of Quetta city, Pakistan.

**Methods:**

A cross-sectional study was conducted at the Obstetrics and Gynecology Department of Sandeman Provincial Hospital Quetta city, Pakistan. The respondents were asked to answer the Urdu (lingua franca of Pakistan) version of the Quality of Life Questionnaire for Physiological Pregnancy. Data were coded and analyzed by SPPS v 21. The Kolmogorov–Smirnov test was used to establish normality of the data and non-parametric tests were used accordingly. Quality of Life was assessed as proposed by the developers. The Chi-square test was used to identify significant associations and linear regression was used to identify the predictors of QoL. For all analyses, *p* < 0.05 was taken significantly.

**Results:**

Four hundred and three pregnant women participated in the study with a response rate of 98%. The mean QoL score was 19.85 ± 4.89 indicating very good QoL in the current cohort. The Chi-Square analysis reported a significant association between age, education, occupation, income, marital status, and trimester. Education was reported as a positive predictor for QoL (*p* = 0.006, β = 2.157). On the other hand, trimester was reported as a negative predictor of QoL (*p* = 0.013, β = −1.123).

**Conclusion:**

Improving the QoL among pregnant women requires better identification of their difficulties and guidance. The current study highlighted educational status and trimester as the predictors of QoL in pregnant women. Health care professionals and policymakers should consider the identified factors while designing therapeutic plans and interventions for pregnant women.

## Introduction

Pregnancy and transition to motherhood is a wonderful experience. For a woman, where pregnancy brings self-fulfillment and indulgence, the fear of the upcoming events in life are also expected (1). As the pregnancy continues, women undergo physiologic, biochemical, and anatomic changes that develop anxiety, depression, and stress in expecting mothers (1). Moreover, motherhood demands adaptation to the intense transformations during the gestational period which is beyond women's control (2). Such changes result in the development of negative body image and dissatisfaction with life that is primarily attributed to the multiple stages of pregnancy (3, 4).

In today's world, the reproductive rights of women are assured in different public policies (5, 6). For that reason, quality of care and provision of services are hospital-centric and usually follow a medicalized and technocratic model (7). However, the least attention is given to non-clinical measures such as changes in mental health, self-esteem and confidence, and Quality of Life (QoL). Therefore, we strongly believe that in addition to standard pharmaceutical care, assessing the above-mentioned factors among pregnant women can provide a strong foundation for maternal health promotion (8).

Correlating, the QoL of pregnant women is influenced by several factors including social insertions, acceptance of gestation, restructuring families, the conception of the mother's role, and preparedness for childbirth during pregnancy (9). All these changes require a pregnant woman to adopt new responsibilities of motherhood. Although changes during the gestational period are short-term, they can remarkably affect the QoL of a pregnant woman (10). Within this context, the World Health Organization defines QoL as “*the position and perception of individuals in life, in the context of value systems and culture they live in with respect to their expectations, goals, concerns and standards*” (11). Moreover, QoL is also influenced by the physical health, psychological state, social relations, and relationship with important elements of the environment of the subject (12). Consequently, healthcare professionals in addition to traditional care must consider changes in QoL that are also supported by literature (13, 14).

In clinical and social research, QoL assessments are used for monitoring outcomes, and multiple instruments are used in literature that are either generic or disease-specific (15). Correlating, Barofsky concluded that definitions and assessment of a comprehensive concept of QoL change with time and objectivity (16). We do agree with this notion as precise methods of assessing QoL are missing in the literature and the ones used still have their limitations (17). Shifting our concerns to QoL in pregnancy, specific tools to assess QoL among pregnant women are least reported in the literature (18). To the best of our knowledge, although the generic WHOQOL-BREF is widely utilized to assess QoL of pregnant women (19), only one instrument i.e., QOL-GRAV is available to depict sensitive and accurate experiences during physiological pregnancy (18). Summarizing the opinions, the relationship between the physiological process of pregnancy and a woman's QoL is least discussed and reported in the literature and that was the major reason for the authors to conduct this study. Also, the study was conducted in a developing country where the QoL of pregnant women in primary care settings is neither reported nor given enough attention as an indicator of improving antenatal care. For that reason, we aimed to develop the profile and predictors of maternal QoL among pregnant women attending a primary healthcare institute of Quetta city, Pakistan.

## Methods

### Study Design

A cross-sectional study was conducted at the Obstetrics and Gynecology Department of Sandeman Provincial Hospital Quetta (SPHQ), Pakistan. Sandeman Provincial Hospital was established in 1939 and is centrally located in the city. Being a public healthcare teaching institute, SPHQ is the facility of choice for most of the population. It has a well-established Obstetrics and Gynecology department with daily consultation of 500 patients per day (20).

### Sample Size and Criteria

The sample size was calculated by the formula proposed by Daniel (21). By keeping the confidence level at 95%, response distribution of 50%, and margin of error at 5%, 377 respondents were calculated. To avoid missing data, a drop out of 10% was added and 414 respondents were conveniently approached for data collection.

Pregnant women attending the Obstetrics and Gynecology Department of SPHQ were approached by the first author. The respondents were informed about the nature of the research and their rights to confidentiality. Respondents not willing to participate, having mental disorders, and immigrants from other countries were excluded from the study.

### Study Instrument

As discussed above, the only specific instrument available for the assessment of QoL in physiological pregnancy is the Quality of Life Questionnaire for Physiological Pregnancy (QOL-GRAV). The tool was developed by Vachkove et al., and the authors concluded that QOL-GRAV can sensitively and accurately capture the experiences of pregnant women that significantly affect their QoL (18). For assessment of QOL we used the validated version of QOL-GRAV-U (22). In addition to the QOL-GRAV-U, we also recorded the demographics of the respondents.

### Ethical Approval

The Institutional Review Board of Faculty of Pharmacy and Health Sciences, University of Balochistan, Quetta approved the study [UoB/Reg/67]. In addition, written consent was also taken from the respondents before the data collection. The participants and attendants/care givers were informed about their rights of participation in the study and the right of withdrawal at any time without compromising their consultation at the healthcare institute.

### Statistical Analysis

The data were coded and entered into SPPS v 21 for formal analysis. The Kolmogorov–Smirnov test was used to establish the normality of the data and non-parametric tests were used accordingly. Frequencies and percentages were used to explain the demographic variables. Quality of Life was measured as proposed by the developers (18). The Chi-square test was used to identify significant associations and linear regression was used to identify the predictors of QoL. For all analyses, *p* < 0.05 was taken significantly.

## Results

### Demographic Characteristics of Study Respondents

Four hundred and three pregnant women participated in the study with a response rate of 98%, whereas 11 participants were dropped out due to the reason that 5 questionnaires had duplicate responses and 6 questionnaires were incomplete. The description of socio-demographic variables and frequency distribution of the respondents are summarized in Table 1. Most of the participants were in age range of 26–35 years (241, 59.8%). Ninety were illiterate, and the cohort was dominated by housewives (75.9%). The majority (91%) had rural residencies and 60.3% were in their second trimester.

**Table 1:** Demographic characteristics of study respondents.

**Characteristics**	**Frequency**	**Percentage**
**Age**		
15–25	116	28.8
26–35	241	59.8
36–45	46	11.4
**Education**		
Illiterate	90	22.3
Metric	71	17.6
Intermediate	84	20.8
Graduation	71	17.6
Post-graduation	87	21.5
**Occupation**		
Housewife	306	75.9
Working women	97	24.1
**Income [Pakistan Rupees=** **Pk. Rs.]**		
None	306	75.9
<10,000	30	7.4
11,000–20,000	31	7.7
>20,000	36	8.9
**Marital status**		
Widowed	5	1.2
Married	398	98.8
**Locality**		
Urban	35	8.7
Rural	368	91.3
**Trimester**		
1	92	22.8
2	243	60.3
3	68	16.8
**Number of children**		
0	10	2.5
1–3	144	35.7
4–6	130	32.3
>6	119	29.5
**Husband's education level**		
Illiterate	79	19.6
Metric	51	12.7
Intermediate	78	19.4
Graduation	90	22.3
Post-graduation	105	26.0
**Husband's occupation**		
Government employee	111	27.5
Private employee	194	48.1
Business	98	24.4

### Assessment of Quality of Life

The QOL-GRAV-U is a 9-item questionnaire where three items out of nine [item 7, 8, and 9] are reverse coded and are presented in a 5-point Likert format. The Likert rating of 1 represents the best and 5 the worst state of QoL. Lower mean scores reflect high QoL and vice versa. According to the developers, QoL is measured as excellent [mean score of 9–18], very good [mean score of 19–27 points], good [mean score of 28–36 points], and not very good [man score of 37–45 points]. In the current study, the mean QoL score was 19.85 ± 4.89 indicating very good QoL in the current cohort (Table 2).

**Table 2:** Quality of Life of the study respondents.

**Items in questionnaire**	**Not at all,** ***N* (%)**	**A little,** ***N* (%)**	**A middle,** ***N* (%)**	**A lot,** ***N* (%)**	**Maximally,** ***N* (%)**
To what extent do you feel that your physical changes associated with this pregnancy do not allow you to do what you need?	119 (29.5)	192 (47.6)	42 (10.4)	41 (10.2)	9 (2.2)
To what extent do you feel that your psychological changes associated with this pregnancy do not allow you to do what you need?	153 (38.0)	159 (39.5)	51 (12.7)	31 (7.7)	9 (2.2)
How worried are you about not being able to handle household chores?	104 (25.8)	119 (29.5)	109 (27.0)	51 (12.7)	20 (5.0)
How worried are you about carrying out the pregnancy successfully?	152 (37.7)	83 (20.6)	51 (12.7)	75 (18.6)	42 (10.4)
How worried are you about not being able to handle labor and delivery?	72 (17.9)	109 (27.0)	74 (18.4)	86 (21.3)	62 (15.4)
Have you been forced to cut down on your physical activity during this pregnancy?	70 (17.4)	90 (22.3)	142 (35.2)	85 (21.1)	16 (4.0)
How satisfied are you with your partner now?	2 (0.5)	5 (1.2)	52 (12.9)	126 (31.3)	218 (54.1)
How satisfied are you with your social life now?	3 (0.7)	11 (2.7)	57 (14.4)	173 (42.9)	159 (39.5)
How satisfied are you with how you manage to adapt to this pregnancy?	24 (6.0)	20 (5.0)	46 (11.4)	134 (33.3)	179 (44.4)

*Based on the total score, the QoL was evaluated as excellent (9–18 points), very good (19–27 points), good (28–36 points), not very good (37–45 points). Mean Quality of Life score in current cohort was 19.85 ± 4.89 indicating very good Quality of Life in the current cohort*.

### Association Between Demographic Variables and Quality of Life

Table 3 presents the cross-tabulation analysis between socio-demographic and study variables. For this analysis, we categorized QoL into good and poor [mean values of >22 as poor and <22 as good QoL]. The Chi-Square analysis reported a significant association between age, education, occupation, income, marital status, and trimester. The interpretation of the significant values reported a moderate strong relationship hence confirming the possibility of linear regression analysis (23). No significant association was reported among other variables.

**Table 3:** Patients' demographics characteristics and Quality of Life.

**Characteristics**	**Quality of Life**		*P*-value^*^	***R*-value**
	**Good**	**Poor**		
**Age**				
15–25	67 (29.9)	49 (27.4)	0.004 (ϕc = 0.386)	0.352
26–35	129 (57.6)	112 (62.6)		
36–45	28 (12.5)	18 (10.1)		
**Education**				
Illiterate	44 (19.6)	46 (25.7)	0.002 (ϕc = 0.386)	0.419
Metric	37 (16.5)	34 (19.0)		
Intermediate	51 (22.8)	33 (18.4)		
Graduation	42 (18.8)	29 (16.2)		
Post-graduation	50 (22.3)	37 (20.7)		
**Occupation**				
Housewife	180 (80.4)	126 (70.4)	0.014 (ϕc = 0.386)	0.377
Working women	44 (19.6)	53 (29.6)		
**Income**				
None	178 (79.5)	124 (69.3)	0.005 (ϕc = 0.286)	0.399
<10,000	8 (3.6)	22 (12.3)		
11,000–20,000	19 (8.5)	12 (6.7)		
>20,000	19 (8.5)	21 (11.7)		
**Marital status**				
Widowed	0 (0)	5 (2.8)	0.017 (ϕc = 0.312)	0.310
Married	224 (100.0)	174 (97.2)		
**Locality**				
Urban	20 (8.9)	15 (8.4)	0.496	0.214
Rural	204 (91.1)	164 (91.6)		
**Trimester**				
1	39 (17.4)	53 (29.6)	0.014 (ϕc = 0.304)	0.425
2	146 (65.2)	97 (54.2)		
3	39 (17.4)	29 (16.2)		
**Number of children**				
0	5 (2.2)	5 (2.8)	0.271	0.214
1–3	78 (34.8)	66 (36.9)		
4–6	81 (36.2)	49 (27.4)		
> 6	60 (26.8)	59 (33.0)		
**Husband's education level**				
Illiterate	43 (19.2)	36 (20.1)	0.262	0.118
Metric	24 (10.7)	27 (15.1)		
Intermediate	44 (19.6)	34 (19.0)		
Graduation	97 (43.3)	67 (37.4)		
Post-graduation	16 (7.1)	15 (8.3)		
**Husband's occupation**				
Government employee	57 (25.3)	55 (30.7)	0.385	0.009
Private employee	111 (49.3)	83 (46.4)		
Business	57 (25.3)	40 (22.3)		

* *Chi square, all entries in bold are significant and values in the brackets are the interpretation of the significant values. R represents the correlation values*.

### Predictor of Quality of Life Among Study Respondents

A simple linear regression was carried out to identify the predictors of QoL in the current cohort. The variables that were significantly associated with QoL were entered into the regression model. The scatter plot showed that there was moderate to strong positive linear relationships between the variables which was confirmed with acceptable Pearson's correlation coefficient (>0.40). Results of the multiple linear regression indicated that there was a collective significant effect between the dependent and independent variables [*F*_(6, 94)_ = 20.82, *p* < 0.001]. Furthermore, the predictors managed to explain 55.5% of the variance (*R*^2^ = 0.55). Education was reported as a positive predictor for QoL (*p* = 0.006, β = 2.157) indicating that for every 1 unit increase in education, there is a possibility that QoL will increase by 2.157 times. On the other hand, trimester was reported as a negative predictor of QoL (*p* = 0.013, β = −1.123) indicating that as trimester increases, QoL decreases by 1.123 as shown in Table 4.

**Table 4:** Factor associated with Quality of Life among pregnant women.

**Model**	**Standardized** **coefficients**	***t***	**Sig**.	**95% confidence interval**	
	**β**			**Lower bound**	**Upper bound**
(Constant)		10.693	0.000	19.006	27.569
Age	0.045	0.911	0.363	−0.417	1.136
Education	2.157	2.759	***0.006***	0.906	0.152
Occupation	0.062	0.583	0.560	−1.684	3.106
Income	0.163	1.454	0.147	−0.280	1.869
Marital status	−0.055	−1.112	0.267	−6.669	1.850
Trimester	−1.123	−2.500	***0.013***	−1.705	−0.204

*Bold and italic values represents significant associations*.

## Discussion

Over the past decades, the assessment of QoL in both clinical and non-clinical settings have gained immense significance. Accordingly, different assessment tools were developed to measure psychological, physical, and social QoL among patients and the general population. However, in comparison to other conditions maternal QoL is least studied in the literature. A possible reason is attributed to the lack of established measures while assessing QoL in pregnant women (18). As a result, the current study was aimed to highlight predictors of maternal QoL in pregnant women of Quetta City, Pakistan. We also did an extensive literature review, and it was revealed that only a few studies report QoL of pregnant women but not from Pakistan.

Our study involved a cohort of 403 pregnant whereby 59% of the respondents were in the age range of 25–35 years. Other studies conducted in Brazil and China also highlighted the similar age group i.e., 26 ± 6.4 and 27.3 ± 4.0 years, respectively (24, 25). The marital status and occupation of women were also like what is reported in Iran (26). However, the other demographics were inconsistent with studies where the literacy rate was higher (24). Large numbers of our respondents (91.3%) were from rural areas and [60.3%] were in the second trimester of pregnancy, unlike the results from a study conducted in France (27). A The possible reason of this differentiation is attributed to the cultural context, marriage age, and practices that vary differently in Pakistan and other regions (especially the developed world).

The mean QoL scores of the current study respondents were 19.85 ± 4.89 which indicated very good QoL. Our results are parallel to what was reported by studies conducted in Serbia and Slovenia (28, 29). The gestational period has a direct relationship with QoL, and this serves as an indicator for health care professionals to take measures for improving QoL (30). Although QoL in the current cohort was promising, we still advocate the provision of special attention and care to pregnant women with poor QoL.

Our study managed to identify various significant relationships between demographics and QoL (Table 3). The published literature does support our results and has established relationships between QoL and demographic characteristics of pregnant women. Age was significantly associated with the maternal QoL in a study conducted by Balíková and BuŽgová in which women of age >29 years had low QoL as compared to younger women (31). In line with what is being discussed; Mazúchová et al. reported the best QoL in younger women when compared with middle-aged women and was worst in the older age group (29). Correspondingly, women with higher educational backgrounds had better QoL (32). We also found that period of pregnancy was also significantly associated with maternal QoL agreeing with the findings of Mazuchova et al. The authors found that the best QoL score was in the first trimester and least in the second trimester of the pregnancy (29). A positive relationship between household income and QoL was also reported (33). However, studies also discovered that there was no relation between QoL and monthly income. This difference is understandable, and the majority of the respondents do not tend to disclose their financial status (32).

A simple linear regression was carried out to identify the predictors of QoL. The variables that were significantly associated with QoL were entered into the regression model. Education was reported as a positive predictor for QoL indicating that for every 1 unit increase in education, QoL improves by 2.157 times. In connection to our findings, a study conducted in Thailand on older pregnant women concluded that education had a direct link with QoL, and women with higher educational status had better QoL (34). A study conducted in Iran revealed that women with higher education status had better QoL than other women (32). Education plays a key role in improved awareness of medical facilities, utilization of healthcare services and understanding of the needs and demands of pregnancy. Educated women are also in a position of getting job offers and can get employment easily as compared to housewives and less educated. Our claims are supported by studies whereby employed pregnant women had improved QoL as job satisfaction and financial stability establish greater self-esteem (9, 10). Concluding, women with higher education have higher self-efficacy and are anticipated to receive encouraging social support therefore have improved QoL.

On the other hand, trimester was reported as a negative predictor of QoL indicating that as trimester increases, QoL decreases by 1.123 times. Zarei et al. also reported similar findings and highlighted gestational age as a negative predictive factor for maternal QoL (26). Zahedi et al., also claimed that with the increase in gestational age, mean scores of QoL decreases. The authors also reported that the maximum score of QoL was found in the first trimester and lowest in the third trimester (35). Conversely, Makvandi et al. did not find any relationship between QoL and gestational age (36). As per our results, best QoL was reported in the first trimester and is attributed to the feeling of parity and happiness of motherhood. In addition, fatigue, nausea, and vomiting starts as pregnancy continues and that decreases QoL. Finally, worse QoL in the third trimester is attributed to an increase in weight, reduced sexuality, and sleep disorders.

Summarizing our results, QoL was very good in most of our respondents. The findings of this study will assist health care professionals in establishing interaction and resolving associated problems that affect QoL during pregnancy. Pregnant women are influenced by various bio-psycho-social factors therefore, it is essential to have a care plan that fulfills the actual needs of this group during the gestational period.

## Conclusion

Improving the QoL among pregnant women requires better identification of their difficulties and guidance. Therefore, frequent assessment of QoL, including its dimensions, of pregnant women can clarify women's health status. In this context, results of this study identified some predictors of QoL that can provide insights for better understanding of factors affecting QoL. Because educational status and trimester appeared as predictors of QoL, healthcare professionals should consider the identified factors while designing therapeutic plans or planned interventions for pregnant women. Despite the positive results, it is crucial to screen the Quality of Life of pregnant women and to pay special care to pregnant women who have a lower Quality of Life.

## Strengths and Limitations

Relationship between pregnancy and woman's QoL is least reported in the literature. Therefore, the current study is pioneer study from Pakistan.

The study was conducted in the Quetta city of Pakistan, which is not representative of the whole country. A comprehensive study is recommended throughout the country to generalize the result. We also recommend that pregnant women visiting private healthcare institutes should be added into the study to underline possible differences of QoL between public and private healthcare facilities.

## Future Recommendations

The study was single cantered, consequently targeting other public and private healthcare institutes may bring diversity of findings. Moreover, assessing QOL in specific trimesters can also bring interesting outcomes. Therefore, a study on different healthcare settings with individualized trimesters is hereby recommended.

## Data Availability Statement

The original contributions presented in the study are included in the article/supplementary material, further inquiries can be directed to the corresponding author/s.

## Ethics Statement

The studies involving human participants were reviewed and approved by Faculty of Pharmacy & Health Sciences. The patients/participants provided their written informed consent to participate in this study.

## Author Contributions

RI, MS, NB, and AS conceptualized and designed the study. AR, SR, FB, and SH collected the data while ZH, FS, and NA analyzed and interpreted the data. The study was supervised by FS, QI, and AHK. All authors have met the criteria for authorship and had a role in preparing the manuscript and also, approved the final manuscript.

## Conflict of Interest

The authors declare that the research was conducted in the absence of any commercial or financial relationships that could be construed as a potential conflict of interest.

## Publisher's Note

All claims expressed in this article are solely those of the authors and do not necessarily represent those of their affiliated organizations, or those of the publisher, the editors and the reviewers. Any product that may be evaluated in this article, or claim that may be made by its manufacturer, is not guaranteed or endorsed by the publisher.
